# A Comparative Study of Spinal Anaesthesia With Hyperbaric Ropivacaine 0.75% and Hyperbaric Bupivacaine 0.5% in Hypertensive Patients Undergoing Lower Abdominal and Lower Limb Surgery

**DOI:** 10.7759/cureus.98437

**Published:** 2025-12-04

**Authors:** Akrity Singh, Ranjeet Rana De, Nitin Kumar, Akhileshwar, Saurav Shekhar

**Affiliations:** 1 Anaesthesiology and Critical Care Medicine, Indira Gandhi Institute of Medical Sciences, Patna, IND; 2 Anaesthesiology (Trauma and Emergency), Indira Gandhi Institute of Medical Sciences, Patna, IND

**Keywords:** bupivacaine, hyperbaric, hypertension, ropivacaine, spinal anaesthesia

## Abstract

Background: Ropivacaine's stereoselective and lipophilic properties contribute to its cardiovascular stability and promote the early recovery of sensory and motor function, facilitating quicker ambulation and discharge. This study aimed to compare the hemodynamic stability measured by blood pressure and heart rate changes of intrathecal 0.75% ropivacaine versus 0.5% bupivacaine in patients with controlled hypertension undergoing spinal anaesthesia.

Methods: Eighty patients classified as American Society of Anesthesiologists (ASA) physical status II and III with controlled hypertension were randomly assigned to either Group R (ropivacaine 0.75%, 3 mL) or Group B (bupivacaine 0.5%, 3 mL). The onset and duration of sensory and motor blockade were recorded, along with incidences of hypotension and bradycardia. Hemodynamic parameters and side effects were monitored at preset intervals during the procedure.

Results: The incidence of hypotension was significantly higher in Group B compared to Group R (p<0.001), while bradycardia was more frequent in Group B but without statistical significance. Motor block duration was 130±30.42 minutes for ropivacaine versus 172±20.80 minutes for bupivacaine; sensory block lasted 160±30.42 minutes and 260±20.40 minutes, respectively.

Conclusion: Hyperbaric ropivacaine 0.75% provides superior spinal anaesthesia in hypertensive patients, with faster sensory and motor recovery (p<0.001), facilitating earlier ambulation and discharge. It also offers enhanced hemodynamic stability compared to equipotent hyperbaric bupivacaine 0.5%.

## Introduction

Local anaesthetics, both esters (e.g., procaine, tetracaine) and amides (e.g., bupivacaine, lidocaine), have long been utilized for spinal anaesthesia. Among them, hyperbaric bupivacaine, commercially available and prepared with dextrose for increased density, remains a widely used intrathecal agent due to its high potency (lipid solubility) and prolonged duration of action. Common side effects include hypotension and bradycardia, primarily caused by the spread of anaesthesia to mid-thoracic spinal levels. Furthermore, the extended motor blockade associated with intrathecal hyperbaric bupivacaine results in undesirable effects, such as urinary retention.

The cardiotoxic potential of intrathecal bupivacaine, notably hypotension and bradycardia, prompted the development of ropivacaine, a pure S-enantiomer of propivacaine. Ropivacaine exhibits reduced cardiotoxicity, shorter anaesthetic duration, and lower lipid solubility [1] compared to bupivacaine. Its lipophilic and stereoselective properties [2] contribute to decreased cardiac and neurotoxicity. Additionally, ropivacaine allows for a faster offset of anaesthesia, producing a preferential sensory blockade over motor blockade. Numerous studies [3] have compared equipotent intrathecal doses of hyperbaric bupivacaine and hyperbaric or isobaric ropivacaine, with and without adjuvants, across various surgical contexts. A key limitation of hyperbaric bupivacaine is the incidence of hypotension, especially in hypertensive patients; intraoperative hypotension has been correlated with elevated risks of stroke and myocardial ischemia. While isobaric ropivacaine is limited by poorly defined spinal anaesthesia spread and hyperbaric ropivacaine is relatively novel, this study aims to compare the anaesthetic profiles of intrathecal hyperbaric ropivacaine 0.75% and hyperbaric bupivacaine 0.5% in patients undergoing lower abdominal and lower limb surgeries.

Controlled hypertension [4] is defined as blood pressure maintained below 140/90 mmHg for more than two weeks via antihypertensive treatment or lifestyle modification.

Hypotension and bradycardia following spinal anaesthesia are common, particularly among hypertensive patients. Although prior research has compared ropivacaine and bupivacaine in spinal anaesthesia, studies specifically involving patients with controlled hypertension are lacking. This study, therefore, seeks to address this gap.

The primary objective is to assess the hemodynamic stability measured by blood pressure and heart rate changes of intrathecal hyperbaric ropivacaine 0.75% versus hyperbaric bupivacaine 0.5% during intraoperative anaesthesia in patients with controlled hypertension undergoing lower abdominal and lower limb surgeries.

## Materials and methods

**Figure 1:**
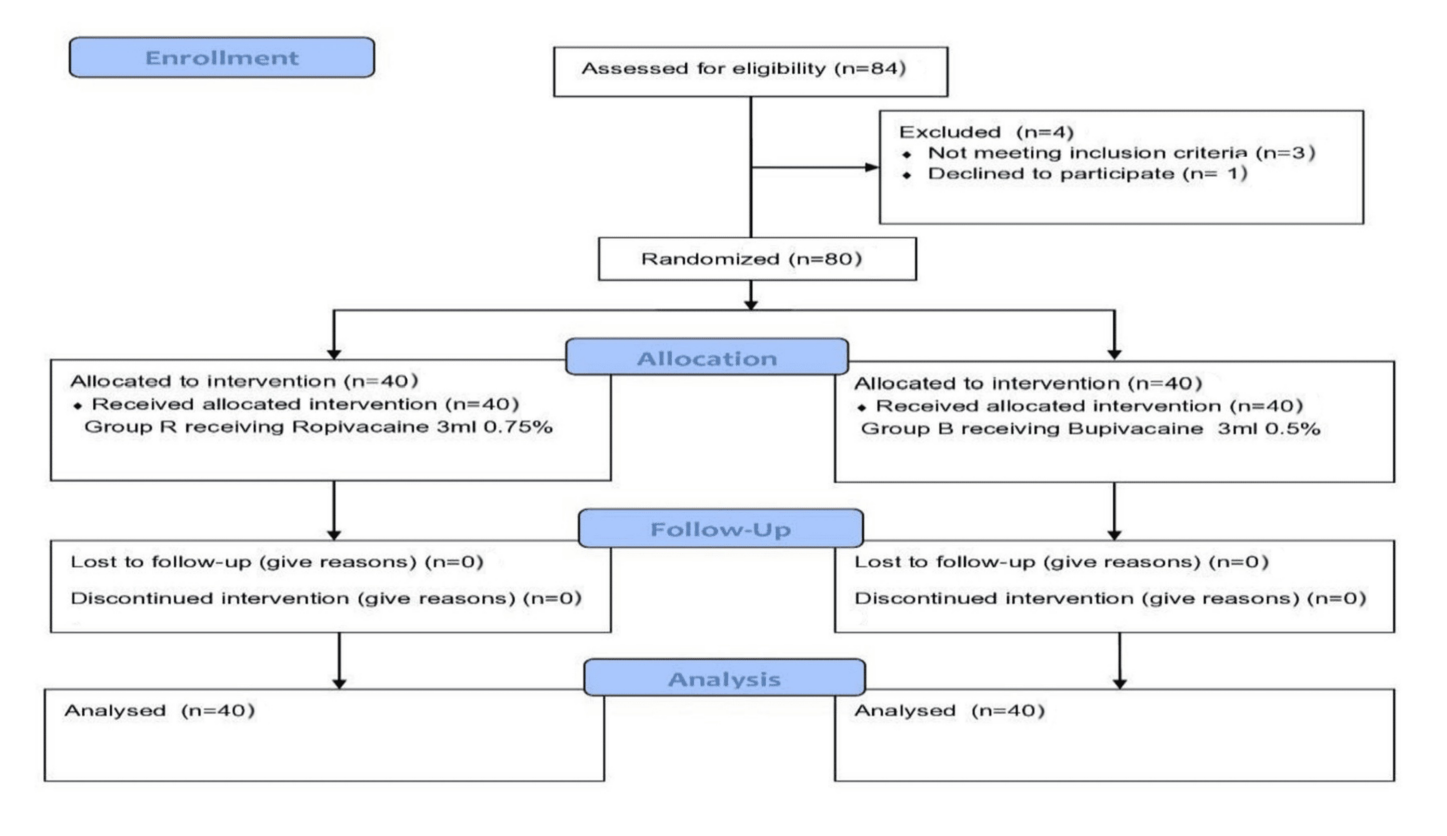
CONSORT diagram showing eligibility and allocation to intervention CONSORT: Consolidated Standards of Reporting Trials

This prospective, randomized, comparative, double-blind controlled study was conducted at Indira Gandhi Institute of Medical Sciences, Patna, India, after obtaining approval from the institute's Institutional Ethics Committee (approval number: 473/IEC/IGIMS/2022; dated: 01.04.2022) and Clinical Trials Registry-India (CTRI) registration (CTRI number: CTRI/2022/06/043360). The study was carried out in accordance with the Code of Ethics of the World Medical Association (Declaration of Helsinki) and the Consolidated Standards of Reporting Trials (CONSORT) statement (Figure 1). Computer-generated random allocation sequence was created. The software created a unique code that is used for each subject for allocation to assign the patients into two groups using coded numbered, opaque, sealed envelopes. Group allocation was performed by an independent anaesthesiologist. Another clinician prepared the syringes containing the study drugs. The patients and evaluators were blinded to the assigned group.

The inclusion criteria were 80 adult patients aged between 25 and 70 years of both sexes scheduled for elective lower abdominal and lower limb surgery with controlled hypertension on drugs under spinal anaesthesia of American Society of Anesthesiologists (ASA) physical status II and III as we are taking hypertensive patients on medication. The exclusion criteria were refusal, emergency surgery, bleeding disorders, being on anticoagulant and antiplatelet therapy, and pregnancy. All patients were evaluated preoperatively, and all primary investigations were done, including hemoglobin, complete blood count, blood urea nitrogen (BUN), serum creatinine, blood sugar, and serum electrolytes sodium and potassium. Electrocardiogram (ECG) findings were recorded. Patients were also educated about the Visual Analogue Scale (VAS) score and instructed on how to express the degree of pain on the scale. All patients were advised to take antihypertensive medicine in the morning of the operation day with sips of water. All antihypertensives were continued until the day of surgery except for diuretics, angiotensin-converting inhibitors (ACEs), angiotensin receptor blockers (ARBs), and long-acting calcium channel blockers. Patients were randomly divided into two groups of 40 each, which was computer-generated: Group R patients received hyperbaric ropivacaine (0.75%) 3 mL, while Group B received hyperbaric bupivacaine (0.5%) 3 mL. 

All patients received tablet ranitidine 150 mg and tablet alprazolam 0.5 mg the night before surgery. A peripheral intravenous (IV) access was performed with an 18G cannula upon arrival at the preanaesthetic room, and injection metoclopramide 10 mg IV and injection ranitidine 50 mg IV was given 1.5 hours before the operation. Preloading with 8-10 mL/kg of Ringer's lactate was done over 10-15 minutes. All patients were asked to void before being transferred to the operation theatre. In the operation theatre, a standard ASA monitor with ECG, non-invasive arterial blood pressure (NIBP), and pulse oximetry (SPO2) were attached, and baseline readings were assessed. Then, in the right lateral position and under aseptic and antiseptic precautions, lumbar puncture was performed using a midline approach at the second and third lumbar intrathecal spaces (L2-3) using a 25G Quincke spinal needle with the bevel-end facing cephalad. The prepared anaesthetic drugs were injected over 10 seconds and given by an independent anaesthetist not involved in the study. Immediately after the intrathecal injection of drugs (taken as 0 minutes), all patients were kept in a supine horizontal position, and readings of blood pressure (BP), heart rate (HR), and mean arterial pressure (MAP) were taken. The degree of motor block onset in the lower limbs was assessed at five minutes, 10 minutes, and 15 minutes using the modified Bromage scale (MBS). The highest level of sensory block attained was checked by the pinprick method. Intraoperative hemodynamic parameters (BP, HR, and MAP) were assessed at one minute, two minutes, three minutes, five minutes, 10 minutes, 15 minutes, 30 minutes, 45 minutes, and 60 minutes. Upon arrival at the postoperative care unit (PACU), postoperative hemodynamic parameters (BP, HR, and MAP) were recorded at 0 minutes, the time taken when patients just arrived at the postoperative recovery unit, and then at 10 minutes, 20 minutes, 30 minutes, and 40 minutes. Regression of motor block in the lower limbs was assessed by using MBS at 0-60-minute, 60-120-minute, and 120-180-minute intervals. Similarly, sensory blockade regression time up to S2 was checked by using the pinprick method in the mid-clavicular line bilaterally. Then, assessments were continued until complete regression of motor block in the lower limbs and sensory block to S2. Hypotension, defined as a fall in systolic blood pressure (SBP) of >20% from the baseline, was treated with IV injection of mephentermine 6 mg or IV fluids or both based on requirements. All patients were catheterized, and the time to the onset of micturition was recorded.

Pain score was assessed using a standard 10 cm linear VAS in the postoperative period every 30 minutes for the first two hours and then at four, eight, 12, and 24 hours. Duration of complete analgesia was defined as the time from intrathecal injection to the time to the first dose of rescue analgesia. Patients were given rescue analgesia with intravenous tramadol 100 mg when VAS ≥3 and then every eight hours. The MBS is described as follows: 0, full movement; 1, inability to raise the extended leg but can bend the knee; 2, inability to bend the knee but can flex the ankle; and 3, no movement. The primary outcome was to evaluate the hemodynamic stability of both drugs, and the secondary outcome was to compare the onset and duration of motor and sensory blockade, the time of rescue analgesic, and the incidence of side effects.

Sample size

The sample size calculation is based on Kulkarni et al. [5], according to which primary outcomes are the incidence of hypotension and bradycardia, and is based on the formula for comparing two independent proportions: $n = \frac{2 (Z_{1-\alpha/2} + Z_{1-\beta})^2 \, p(1 - p)} {d^2}$. Here, n is the required sample size per group, $Z_{1-\alpha/2}$ is the standard normal deviate corresponding to the desired confidence level (e.g., 1.96 for 95% confidence), $Z_{1-\beta}$ is the standard normal deviate corresponding to the desired power (e.g., 0.84 for 80% power), p is the average (pooled) proportion, and d is the absolute difference between the two proportions to be detected, which comes out to be 38 per group. After adding 10% to the calculated sample size to account for dropouts or protocol deviations, we are taking 40 patients in each group total of 80 patients.

Statistical analysis

The data collected in this study were analyzed by using IBM SPSS Statistics for Windows, Version 22.0 (IBM Corp., Armonk, New York, United States). Microsoft Excel (Microsoft Corporation, Redmond, Washington, United States) was used for data entry. Numerical variables were presented as mean and standard deviation for patient characteristics such as age, weight, height, hemodynamic changes, block parameters such as onset, duration, and recovery time of sensory block, time to maximum motor blockade, duration of motor blockade, and time to first micturition. Categorical variables such as sex distribution, ASA physical status, type of surgery, Bromage grade of motor blockade, and incidence of adverse events, including hypotension and bradycardia, were presented as frequency and percentage where appropriate. P<0.05 was considered statistically significant. Student's unpaired t-test was used for comparisons of mean and proportion.

## Results

Patients in both study groups were not different regarding their initial demographic data (Table 1). They were similar in age, weight, height, and sex distribution, and the data were insignificant. There were no exclusions during the study, and no patients were converted to general anaesthesia.

**Table 1:** Patients' demographic data in both groups The demographic table shows no significant difference between the groups. The values are expressed as mean±SD (age, weight, height, BMI, duration of surgery), numerical (sex), and percentage (ASA grade). Significance was set at p<0.05, computed using an independent t-test for continuous variables and the chi-squared test or Fisher's exact test for categorical variables. ASA: American Society of Anesthesiologists

		Group R (n=36), mean±SD	Group B (n=39), mean±SD	P-value
Age (years)		38.6±8.86	38.70±7.45	>0.05
Weight (kg)		79.5±13.6	76.2±12.8	>0.05
Height (cm)		165.5±5.5	164.2±6.2	>0.05
BMI		29.0±4.3	28.3±4.5	>0.05
				Percentage (%)
Sex		Male: 24	Male: 27	40
		Female: 16	Female: 13	32
ASA	II (%)	27 (90)	26 (86.7)	1.0
	III (%)	3 (10)	4 (13.3)	
Total duration of surgery		96.75±35.84	87.00±42.25	0.691

Table 2 shows the onset and duration of motor blockade with bupivacaine and ropivacaine with early onset and prolonged duration of motor and sensory action of bupivacaine.

**Table 2:** Onset and duration of motor blockade with bupivacaine and ropivacaine in both groups The values are expressed as mean±SD and 95% CI. Significance was set at p<0.05.

Parameters	Group B	Group R		P-value
Sensory block onset at T10 (min)	3.0±1.2		6±1.8	<0.001
Total duration (min)	260±20.40 (95% CI: 253.67-266.32)		160±30.40 (95% CI: 150.57-169.42)	<0.001
Motor block onset (min)	9±2.0		13±2.4	<0.001
Total duration (min)	172±20.80 (95% CI: 165.54-178.44)		130±30.42 (95% CI: 120.57-139.42)	<0.001

The total fluid load administered, the amount of blood loss, and the duration of surgery were statistically not significant between the two groups at the first hour.

At one minute, Group R had an SBP of 136.4±6.8 mmHg, slightly higher than Group B's SBP of 134.8±7.2 mmHg, with no statistically significant difference (p=0.31). At five minutes, SBP significantly dropped to 115.28±7.2 mmHg with a 95% CI of 112.98-117.58 in Group R but was much lower at 90±8.4 mmHg with a 95% CI of 87.31-92.69 in Group B, showing a highly significant difference (p<0.001) (Table 3).

**Table 3:** SBP at different points of time in both groups The data are expressed as mean±SD and 95% CI. Significance was set at p<0.05. SBP: systolic blood pressure

Time	Group R SBP (mmHg)	95% CI of Group R	Group B SBP (mmHg)	95% CI of Group B	P-value
1 min	136.4±6.8	134.23-132.50	134.8±7.2	132.50-137.10	0 .31
5 min	115.28±7.2	112.98-117.58	90±8.4	87.31-92.69	<0.001
10 min	127.01±9.33	124.03-129.99	121.48±8.24	118.85-124.11	0.006
15 min	121.61±7.7	119.15-124.07	112.16±8.84	109.33-114.99	<0.001
20 min	119.32±6.4	117.27-121.37	110.60±10.28	107.31-113.89	<0.001
30 min	116.60±4.7	115.10-118.10	116.42±9.76	113.30-119.54	0.917
45 min	115.2±7.3	112.87-117.53	114.92±6.54	112.83-117.01	0.857
60 min	118.6±5.5	116.84-120.36	114.34±8.12	111.74-116.94	0.008

**Figure 2:**
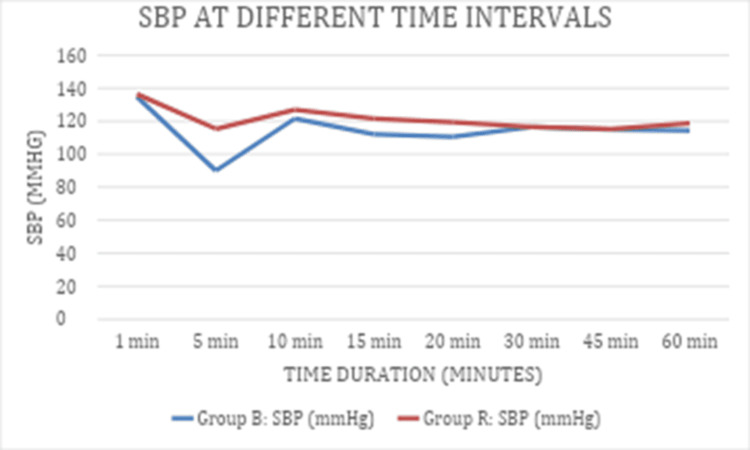
SBP at different time intervals in both groups SBP: systolic blood pressure

This pattern indicates that, except for the early and late time points, SBP was significantly higher in Group R compared to Group B during the first 20 minutes post-measurement, with convergence between groups at the intermediate time points (Figure 2).

**Figure 3:**
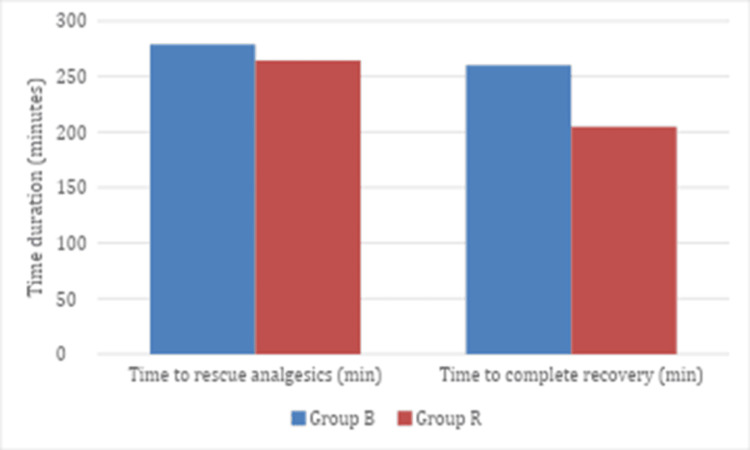
Time to complete recovery and time to rescue analgesic in both groups

The mean time to rescue analgesic administration was 278.75 minutes with a standard deviation of 57.46 and a 95% CI of 260.94-2966.55 in Group B, whereas Group R had a mean time of 264.25 minutes with a standard deviation of 41.11 and a 95% CI of 251.51-276.99. Regarding recovery, the mean time to complete recovery in Group B was 260 minutes±40.78, with a 95% CI of 247.36-272.63, while in Group R, it was 204.75 minutes±34.39, with a 95% CI of 194.09-215.40. These results indicate that both the time to rescue analgesic and the time to complete recovery were longer in Group B compared to Group R, as reflected by the respective means and standard deviations reported (Figure 3).

**Figure 4:**
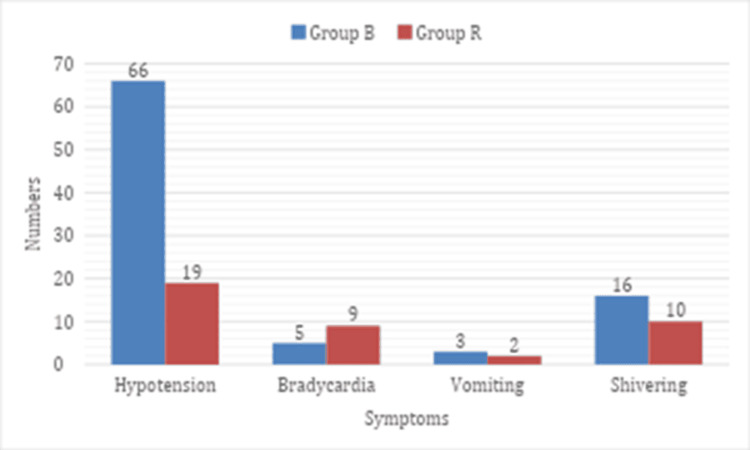
Incidence of side effects following spinal anaesthesia in both groups

The bar graph in Figure 4 demonstrates that among patients in Group B, there were 66% cases of hypotension, while Group R had 19% cases of this condition. Bradycardia occurred in five patients from Group B and in nine patients from Group R. Regarding postoperative side effects, vomiting was reported in three individuals from Group B and in two individuals from Group R. Shivering was observed in 16 patients in Group B compared to 10 in Group R.

## Discussion

The sooner the recovery, mobility, and discharge after surgery, the better the clinical outcome. Early postoperative recovery, enhanced mobility, and prompt discharge optimize clinical outcomes. Our findings indicate that intrathecal hyperbaric ropivacaine 0.75% provides superior anaesthesia with accelerated recovery, enabling earlier ambulation and discharge compared to hyperbaric bupivacaine 0.5%. This aligns with Kulkarni et al. [5], who reported that ropivacaine 15 mg in 8.3% dextrose offers shorter action duration and better anaesthesia than bupivacaine 15 mg in 8% dextrose.​

Conversely, Dar et al. [6] observed faster sensory block recovery and prolonged analgesia with hyperbaric bupivacaine versus ropivacaine. Consistent with this, our study demonstrated quicker onset and extended motor blockade with bupivacaine 0.5% compared to ropivacaine 0.75% (Table 2). These differences underscore the need for agents balancing efficacy, safety, reduced side effects, and rapid recovery; rescue analgesia and complete recovery times were longer in the bupivacaine group (Figure 3).​

Khundongbam et al. [7] found hyperbaric ropivacaine 15 mg with fentanyl 20 μg superior to bupivacaine 10 mg (heavy) for block characteristics and hemodynamic stability. Similarly, our ropivacaine group exhibited better hemodynamic profiles (Figure 2).​

Whiteside et al. [8] reported shorter sensory regression to T10 with hyperbaric ropivacaine (56.5 (28-145) min) versus bupivacaine (118 (80-238) min) using 3 mL of 0.5% solutions in lower abdominal/perineal/limb surgeries. Singh et al. [9] noted faster motor recovery (Bromage 0) with ropivacaine (127±20.42 min) than bupivacaine (182±30.83 min), mirroring our results: ropivacaine 130±30.42 min (95% CI: 120.57-139.42) versus bupivacaine 172±20.80 min (95% CI: 165.54-178.44).​

Gebrargs et al. [10] showed that controlled hypertensive patients develop more hypotension under spinal anaesthesia than normotensives, without bradycardia differences. We observed greater SBP reductions at 5-20 min with bupivacaine versus ropivacaine, but similar heart rates (Figure 2 and Figure 4). This may stem from heightened sympathetic/norepinephrine activity, reduced parasympathetic tone, vascular stiffness [11], and perioperative nifedipine in hypertensives.​

Kalbande et al. [12] and Mahajan and Patel [13] confirmed ropivacaine 0.75% as a safer alternative to bupivacaine 0.5%, with faster motor recovery and comparable spread/duration. For elective regional anaesthesia in hypertensives, ropivacaine 0.75% offers effective anaesthesia, rapid motor return, and superior stability over cardiotoxic bupivacaine, which is similar to the outcomes of our study.​

The study limitations include the small sample size, the variable antihypertensive drugs across groups, and its single-center design.

## Conclusions

Hyperbaric ropivacaine 0.75% turned out to be safer in hypertensive patients in comparison to hyperbaric bupivacaine 0.5% undergoing surgeries under spinal anaesthesia. The onset and recovery from anaesthesia were early in the ropivacaine group as compared to the bupivacaine group (p<0.001). The hemodynamic changes in reference to a fall in SBP were seen more in the bupivacaine group (p<0.001) after the intrathecal administration of drugs. The incidences of side effects were not significant between the two groups.
